# Enhancing the antimicrobial activity of natural extraction using the synthetic ultrasmall metal nanoparticles

**DOI:** 10.1038/srep11033

**Published:** 2015-06-05

**Authors:** Huanhuan Li, Quansheng Chen, Jiewen Zhao, Khulal Urmila

**Affiliations:** 1 School of Food and Biological Engineering, Jiangsu University, Zhenjiang 212013, P.R. China

## Abstract

The use of Catechin as an antibacterial agent is becoming ever-more common, whereas unstable and easy oxidation, have limited its application. A simple and low-energy-consuming approach to synthesize highly stable and dispersive Catechin-Cu nanoparticles(NPs) has been developed, in which the stability and dispersivity of the NPs are varied greatly with the pH value and temperature of the reaction. The results demonstrate that the optimal reaction conditions are pH 11 at room temperature. As-synthesized NPs display excellent antimicrobial activity, the survival rates of bacterial cells exposed to the NPs were evaluated using live/dead Bacterial Viability Kit. The results showed that NPs at the concentration of 10 ppm and 20 ppm provided rapid and effective killing of up to 90% and 85% of *S. aureus* and *E. coli* within 3 h, respectively. After treatment with 20 ppm and 40 ppm NPs, the bacteria are killed completely. Furthermore, on the basis of assessing the antibacterial effects by SEM, TEM, and AFM, it was found the cell membrane damage of the bacteria caused by direct contact of the bacteria with the NPs was the effective mechanism in the bacterial inactivation.

Infectious diseases induced by pathogens bacteria continue to be one of the greatest health problems worldwide, afflicting millions of people annually^1^. *Escherichia coli (E. coli)* and *Staphylococcus aureus* (*S. aureus*) are major bacterial pathogens that can cause life-threatening human diseases^2^,^3^. Recently emerged strains of *E. coli* and *S. aureus* show increased virulence and enhanced ability to cause disease in otherwise healthy individual^4^,^5^,^6^. Herein, antibacterial materials are widely used to target pathogens bacteria in daily life and effectively protect the public health^7^. A series of drug of materials, including antibiotics, metal ions, and quaternary ammonium compounds, can inhibit bacteria growth and destroy cellular structure of microorganisms. However, it is known that the above materials are associated with concerns about antibiotic resistance, complex chemical synthesis, environmental pollution, and high cost^8^,^9^. More recently, natural antibacterial agents have been explored to overcome these disadvantages. Among these natural antibacterial agents, Catechin is a non-toxic, cheaper and naturally broad-spectrum antibacterial agent^10^. Bacterial species has been described included *Escherichia coli, Stenotrophomonas maltophilia, Bacillus bacteria, Listeria monocytogenes, and Staphylococcus aureus.* Unfortunately, Catechin, which can easily damage cells, is unstably prone to the oxidation-reduction reaction^11^, the application of it is limited. In order to overcome this demerit, it is important to control the interaction between the Catechin to prohibit the oxidation.

Herein, we developed here a novel Catechin-Cu nanoparticles(NPs) based on the chelating of Cu(II) to Catechin to overcome the limitation of Catechin. Nowdays, nanotechnology has turn into a keyword of public interest, and a part of our daily life, as the social and economic impact of nanotechnological developments is being recognized^12^. However, there are still several unknown aspects of the widespread application of the nanosciences in human life, in the fields including novel materials manufacturing, electronics, cosmetics, pharmaceutics, and medicine^13^. During the past decade, the application of nanomaterials in medicine significantly increased, which resulted in raising hopes for employing NPs as alternative antibacterial agents. NPs, with at least one of its dimension in the range of 1–100 nm, large surface area, excellent properties^14^,^15^,^16^, have drawn much attention. NP has been increasingly applied to the development of novel antibacterial agents for the management of pathogenic bacteria affecting agricultural crops, humans, and animals. In particular, significant development in nanopaticles synthesis, such as polymeric and metallic, has attracted researchers’ attention toward applications in managing infection diseases caused by bacteria^17^. Therefore, there has always been a strong driving force to develop a simple and low-cost approach to prepare a novel Catechin-Cu nanoparticle which can not only overcome the limitation of Catechin but also target the pathogenic bacterium efficiently. The antimicrobial activities of Catechin-copper (II) complexes have been attempted, but mostly use the monomers of Catechin such as EGCG, EGC, ECG and EC as the raw material^18^,^19^. In contrast to Catechin, these monomers cannot have the wide range of application due to their expensive price. In addition, Catechin-copper (II) complexes are difficult to exhibit strong antimicrobial activity because of their bulk morphology.

In this study, we selected the much cheaper catechin and cuprate as the raw materials, synthesized ultrasmall Catechin-Cu nanoparticles with a simple and low-energy-consuming ultrasonic crushing approach. Then pathogenic bacterium *S. aureus* and *E. coli* were used as model bacterial species to evaluate the antibacterial activity of the Catechin-Cu nanoparticles. In depth, the particle size, surface charge of the materials, antibacterial efficiency, changes in cellular morphology, as well as the contacting degree between the material and the bacteria were all studied. It is worth noting that we first provide the comprehensive experimental data to compare the antibacterial efficiency of Catechin and Catechin-Cu nanoparticles, which shows that this NP at the concentration of 10 mg L^−1^ (10 ppm) and 20 mg L^−1^ (20 ppm) provided rapid and effective killing of up to 90% and 85% of gram-positive *S. aureus* and gram-negative *E. coli* bacteria within 3 h, respectively. After treatment with 20 ppm and 40 ppm NPs, the *E. coli* and *S. aureus* are killed completely within 3 h. NPs demonstrated better antibacterial efficiency than Catechin. Taken together, we expect that this study can offer significant reference for the selection of Catechin-Cu nanoparticles in the real application and the aspect researching of Catechin oxidation.

## Results

### Catechin-Cu nanoparticles characterization and stability

**Figure 1:**
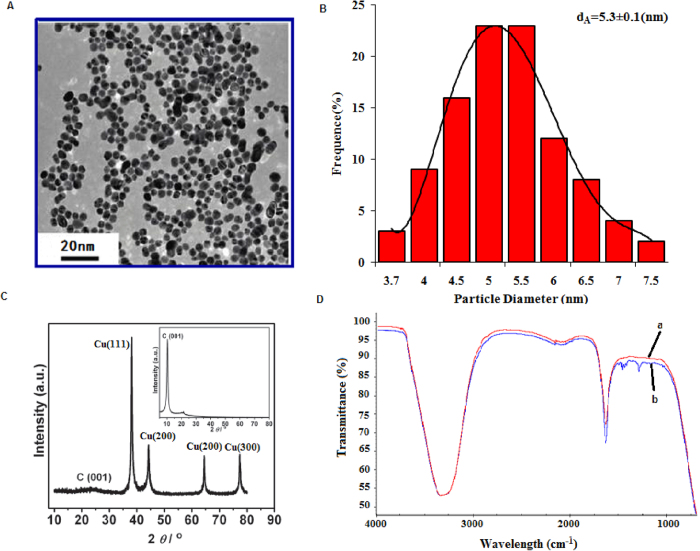
The characterization of Catechin-Cu nanoparticles. (**Panel A**) TEM images of Catechin-Cu nanoparticles. (**Panel B**) The size distribution of Catechin-Cu nanoparticles.d_A_ = 5.3 ± 0.1(nm). (**Panel C**) Typical XRD pattern of the obtained Catechin-Cu nanoparticles; inset: the pattern for Catechin. (**Panel D**) IR spectra of Catechin-Cu nanoparticles (**a**) and Catechin (**b**).

Both Catechin and cupric nitrate compounds showed the capability to spontaneously self-assemble as Catechin-Cu nanoparticles(NPs) in water by ultrasonic crushing method. Transmission electron microscopy (TEM) analysis was performed on a Catechin-Cu nanoparticles suspension and revealed a spherical and regular shape of the NPs (Fig. 1A) and the size distribution of Catehcin-Cu nanoparticles were determined by dynamic light scattering (DLS) (Fig. 1B). As shown in Fig. 1B. Catehcin-Cu nanoparticles have a normal size distribution with an average diameter of 5.3 ± 0.1 nm. Additionally, the zeta potential data (+36.60 mV) demonstrated a positive surface charge for the NPs. According to zeta potential analysis theory, zeta potential data was +36.60 mV corresponding to a large plenty of positive charge on the surface of the Catechin-Cu NP. As a result, it can absorb negative bacteria cell to damage it. Fig. 1C shows the X-ray diffraction (XRD) pattern of the Catechin-Cu nanoparticles, in which reflections of Catechin-Cu nanoparticles are identified. The Catechin-Cu nanoparticles characteristic absorbance peaks at 38.1, 44.3, 64.4, and 77.4° with high intensity, which can be assigned to the (111), (200), (211) and (300) planes of the cubic Cu crystal (JCPDS No.85-1326). Compared with Catechin (see inset of Fig. 1C), the characteristic peak of C(001) shifted from 10.3 to 22.4°, thereby suggesting the reduction of Catechin. However, the diffraction peaks of Catechin are relatively weak in the as synthesized nanoparticles. We conclude that a kind of Catechin-Cu nanoparticle have been formed during this simple and low-energy-consuming method. Fig. 1D shows a peak at 1650 cm^−1^ that is assigned to the stretching vibration of the C = C in the Catechin. Comparing the spectra of Catechin and Catechin-Cu nanoparticles (Fig. 1D(a,b)), the curve of Catechin-Cu nanoparticles represented a peak at 1286.34 cm^−1^ and 1431.29 cm^−1^ are supposed to be the reaction of Catechin and Cu(II). The peak at 1286.34 cm^−1^ that is assigned to the stretching vibration of the C-O in the Catechin-Cu nanoparticles. The peak at 1431.29 cm^−1^ was also found in the spectrum, associated with the stretching vibration of heteroaromatics. This result indicates that the occurrence of chelating reactions during the fabrication process of Catechin-Cu nanoparticles.

**Figure 2:**
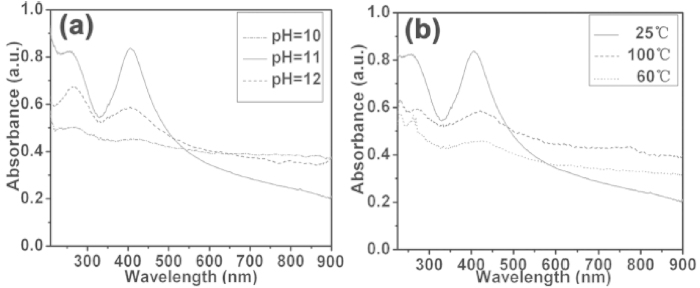
Typical UV-visible spectra of the Catechin-Cu nanoparticles synthesized at different a) pH values and b) reaction temperatures.

It is well known that the optical properties of Catechin-Cu nanoparticles are strongly dependent on their size and shape, and the structure of the nanoparticles can be described by using the UV/Vis spectra. The typical UV/Vis spectra of the Catechin-Cu nanoparticles synthesized under different pH values at room temperature for 70 min are shown in Fig. 2a. The nanoparticles exhibited one characteristic absorbance peak at 420 nm, which was the absorbance peak of Catechin-Cu nanoparticle. Under a pH value of 10, the absorbance peak of the NPs appeared at 420 nm. As the pH was mediated to 12, the clear absorbance peak shifted from 420 to 409 nm, and the full width at half maximum (fwhm) of the spectrum was 82 nm. This fwhm is wide, possibly due to the large size and wide partice size distribution of the NPs. When the pH value was mediated to 11, the narrow nature of the observed peak blue-shifted to 403 nm, and the fwhm decreased to 64 nm, which also suggested the decreasing size and little variation in particle size of the Catehcin-Cu nanoparticles. Fig. 2b shows the UV/Vis spectra of Catechin-Cu nanoparticles synthesized under a reaction temperature of 25, 60, and 100° at the pH value of 11. When the temperature was raised, the absorption of Catechin-Cu nanoparticles became weaker, and the clear absorption peak red-shifted. The wider fwhm suggests the increasing size and polydispersivity of the Catechin-Cu nanoparticles. The results indicated that the stability and dispersivity of Catechin-Cu nanoparticles are better under a room reaction temperature at the pH value of 11.

### MICs and MBCs

In this study, the MIC and MBC values of Catechin and Catechin-Cu nanoparticles were evaluated by broth microdilution method^20^,^21^. Table 1 shows the MIC and MBC of Catechin and Catechin-Cu nanoparticles against various microorganisms. According to these data, the antibacterial activity of Catechin-Cu nanoparticles is significantly higher than that of Catechin. Furthermore, these results can suggest that the Catechin-Cu nanoparticles show the excellent antibacterial activity and against Gram-negative bacteria (*E. coli*) were significantly weaker than that against Gram-positive bacteria (*S. aureus*). Moreover, the MIC and MBC values of Catechin-Cu nanoparticles against some bacteria are lower than those of Catechin, which indicate higher antibacterial activity. After 3 h, the bacteria in control medium grew into the stationary phase, whereas 20 ppm Catechin-Cu nanoparticles showed a significant growth inhibition effect against *E. coli*, and 10 ppm for *S. aureus*. As the concentration of Catechin-Cu nanoparticles increased to 40 ppm and 20 ppm, the *E. coli* and *S. aureus* cells were killed completely within 3 h. The kill ratio of Catechin-Cu nanoparticles against cells as much as 99.99%.

### Bactericidal efficiency

**Figure 3:**
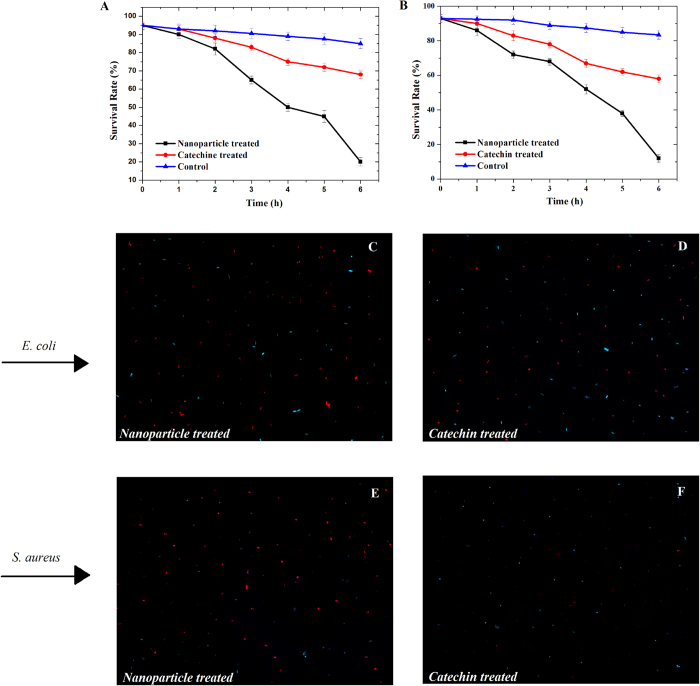
Live /Dead BacLight viability results and fluorescence images. (**A**) The survival rates of *E. coli* with different treatments (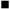) nanoparticles (20 ppm) treated, (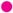) Catechin (20 ppm) treated, and (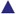) Control. (**B**) The survival rates of *S. aureus* with different treatments (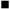) nanoparticles (10 ppm) treated, (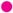) Catechin (10 ppm) treated, and (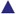) Control. The error bars indicate standard deviations. The representative fluorescence images of *E. coli* (top row) and *S. aureus* (bottom row) by different treatments. (**C**) Fluorescence image of *E. coli* treated with Catechin-Cu nanoparticles (20 ppm). (**D**) Fluorescence image of *E. coli* treated with Catechin (20 ppm). (**E**) Fluorescence image of *S. aureus* treated with Catechin-Cu nanoparticles (10 ppm). (**F**) Fluorescence image of *S. aureus* treated with Catechin (10 ppm). *E. coli* and *S. aureus* all stained by the live/dead staining kit. The green and red fluorescence images are overlaid to one picture for better comparison of living and dead cells.

In the Live/Dead Baclight assay, survival rate is defined in terms of preservation of membrane integrity as the percentage of bacterial cells with intact membranes (stained with SYTO-9) to the total number of cells. The bacterial survival rates (%) of Catechin and Catechin-Cu nanoparticles within 3 h against *E. coli* and *S. aureus* were qualified (Fig. 3A,B). The results indicate that suspended bacteria remained mostly viable over a period of 3 h, whereas the survival rates of *E. coli* expose to Catechin and Catechin-Cu nanoparticles declined to 49% and 15% within 3 h and declined to 42% and 10% for *S. aureus.* Although previous studies have also researched the antibacterial activity of Catechin and NPs, those studies have not attempted to quantify the loss in microbial viability with time; namely, the bacterial survival rate. Meanwhile, they don’t attempt to compare the antibacterial efficacy of Catechin and Catechin-Cu nanoparticles against gram-positive bacterium (*E. coli*) and gram-negative bacterium (*S. aureus*), respectively. Fig. 3C,D show the fluorescence pictures of *E. coli* cells expose to Catechin-Cu nanoparticles and Catechin as well as the corresponding *S. aureus* in Fig. 3E,F. The green fluorescence caused by SYTO 9 is the live cells and dead cells fluoresce in red. As shown in Fig. 3C,E, Catechin- Cu nanoparticle treated bacteria were almost red with fluorescence, indicating the highly permeability of PI dye toward NPs treated bacteria, and most of the cells were dead. The Catechin treated bacteria show strong green fluoresce (Fig. 3D,F), revealing that Catechin had a weak destructive effect on the cell membranes and majority of bacteria were viable. These results demonstrated that the antibacterial efficacy of Catechin-Cu nanoparticles was significantly higher than that of Catechin. This can be attributed to the advantages of NPs and the limitation of Catechin. Catechin- Cu nanoparticle had a size of 5.3 ± 0.1 nm that can be easier attach to the surface of bacteria^22^ and the positive surface charge on Catechin- Cu nanoparticles facilitates a direct interaction between the particles^23^ and the negatively charged bacteria membranes. In addition, *S. aureus*, a strain representing Gram-positive bacteria, exhibited a lower survival rate in comparison to the *E. coli* strain and was hence lower resistant to the antibacterial effect of the Catechin- Cu nanoparticles. These results support the previous assumption that *E. coli* is more tolerant than *S. aureus* to Catechin-Cu nanoparticles.

### Cell integrity disruption of bacteria induced by Catecin-Cu nanoparticles

**Figure 4:**
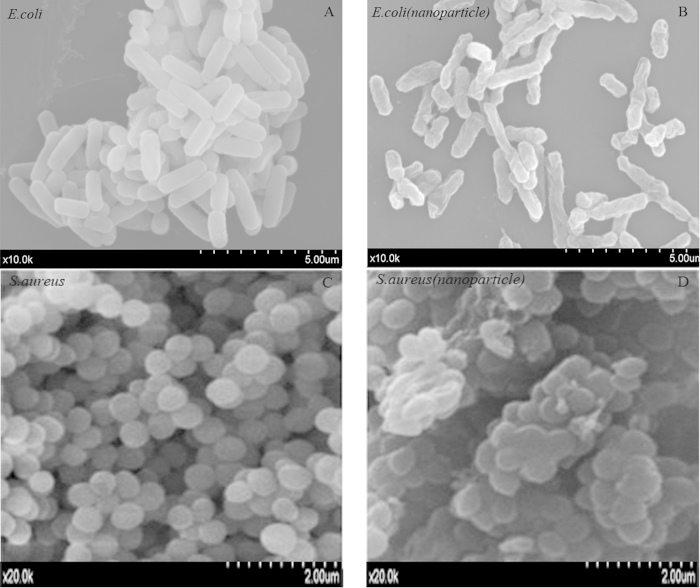
Typical scanning electron microscope (SEM) images. (**A**) Untreated *E. coli*. (**B**) Catechin-Cu nanoparticles (20 ppm) treated *E. coli*. (**C**) Untreated *S. aureus*. (**D**) Catechin-Cu nanoparticles (10 ppm) treated *S. aureus*.

The Catechin-Cu nanoparticles could effectively inhibit the growth of *E. coli* and *S. aureus*. We therefore investigated the morphology changes of *E. coli* and *S. aureus* cells before and after exposure to the Catechin-Cu nanoparticles by scanning electron microscope (SEM), transmission electron microscope (TEM), and atomic force microscope (AFM). The obtained SEM micrographs of *E. coli* and *S. aureus* cells were shown in Fig. 4. As shown in Fig. 4A,C, untreated *E. coli* and *S. aureus* were typically rod-shaped and round-shaped, respectively, both with smooth and intact cell walls. After exposure to Catechin-Cu nanoparticles for 3 h, the quantity of *S. aureus* greatly decreased and cell walls became wrinkled and damaged, similar to previous reports. The shape and size of cells also damaged dramatically and there were a lot of materials attached on the bacterial surface (Fig. 4D). As for *E. coli*, the morphologies of most of the survival cells remained unchanged with round-shape and smooth surface (Fig. 4B). It is likely that Catechin-Cu nanoparticles exhibited different impacts on the G- and G+ bacteria. The results provide further evidence of the damage of the bacterial membrane of G- and G+ bacteria.

**Figure 5:**
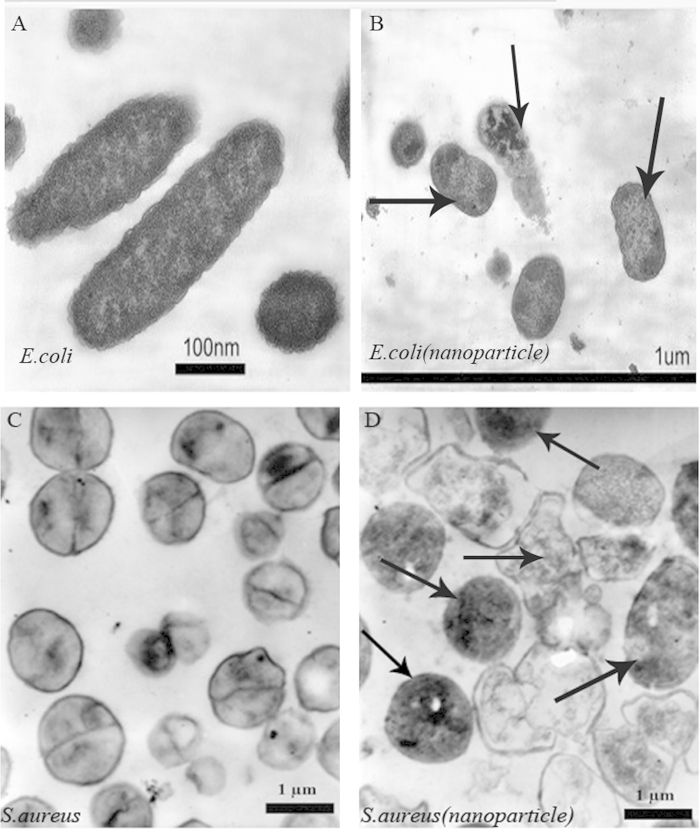
Morphological comparison of bacteria by TEM imaging. (**A**) Untreated *E. coli*. (**B**) Catechin-Cu nanoparticles (20 ppm) treated *E. coli*. (**C**) Untreated *S. aureus*. (**D**) Catechin-Cu nanoparticles treated (10 ppm) *S. aureus* (The images B and D were taken at the MIC of Catechin-Cu nanoparticles).

To investigate the interaction, we decided to perform a TEM characterization of bacteria cultures exposed to Catechin-Cu nanoparticles, in order to find additional evidence of the different effect observed. The TEM images obtained showed that the NPs are present inside the cytoplasm of *S. aureus*, associated with large zones of translucent cytoplasm, featuring either localized or complete separation of the cell membrane from the cell wall (Fig. 5D). Conversely, as for *E. coli*, the NPs do penetrate into the cellular wall, and no evident damage in some *E. coli* is observed close to the NPs (Fig. 5B). Obviously, *E. coli* presented a strong resistant to the antibacterial effect of the Catechin- Cu nanoparticles in comparison to the *S. aureus* strain. The TEM results are in agreement with the results using SEM analysis.

**Figure 6:**
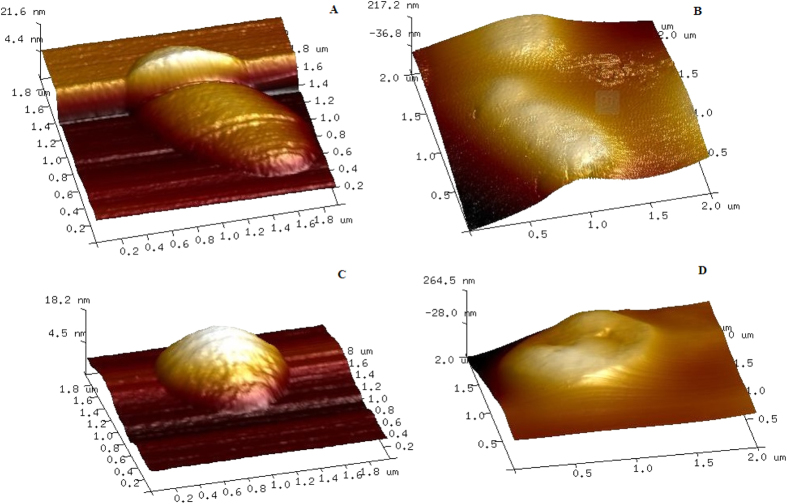
Tapping-mode atomic force microscopy (AFM) images. (**A**) Untreated *E. coli*. (**B**) Catechin-Cu nanoparticles(20 ppm) treated *E. coli* (**C**) Untreated *S. aureus*. (**D**) Catechin-Cu nanoparticles (10 ppm) treated *S. aureus* (scan field area 2.0 μm × 2.0 μm).

Fig. 6 shows the representative AFM images of untreated *E. coli* and Catechin-Cu nanoparticles treated *E. coli.* Untreated *S. aureus* and Catechin-Cu nanoparticles treated *S. aureus.* Initially *E. coli* bacteria appeared as intact rods with no evidence of membrane rupture and collapse (Fig. 6A). However, upon treatment with Catechin-Cu nanoparticles, the bacteria demonstrated strong evidence of membrane disorganization with greater roughness as compared to the smooth surface of untreated *E. coli* (Fig. 6B). Similarly, treated *S. aureus* (Fig. 6D) showed profound membrane damage and distortion with increased roughness contrary to the smooth contour of the control *S. aureus* (Fig. 6C). Aiming to certify about the existence of differences between membranes before and after Catechin-Cu nanoparticles treated, at nanometric level, roughness values (Rq, Ra) were obtained (Table 2). It is showed clearly that after Catechin-Cu nanoparticles treated, the surface roughness of *E. coli* increased from 6.32 nm, 4.88 nm (Rq, Ra) to 71.6 nm, 57.3 nm (Rq, Ra) and from 4.51 nm, 3.13 nm (Rq, Ra) to 76.1 nm, 59.9 nm (Rq, Ra) for *S. aureus*. The increase in surface roughness of bacteria suggests the existence of membrane damage^24^,^25^. We can notice that the change of surface roughness of *S. aureus* (71.59, 56.77) is greater than that of *E. coli* (65.28, 52.42). These results were in accordance with the SEM and TEM results. Taken together, these SEM, TEM and AFM images are clear evidence for the antibacterial effects of Catechin-Cu nanoparticles.

### Cytotoxicity of Catechin-Cu NPs and Catechin

**Figure 7:**
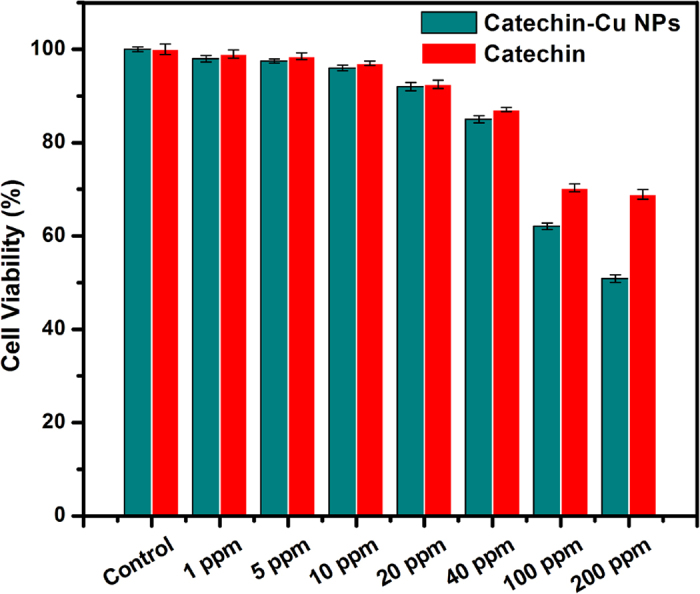
Cytotoxicity of Catechin-Cu nanoparticle and Catechin in HepG2 cells as assessed by MTT assay. Cells were treated with silica nanoparticles at the concentrations of 0, 1, 5, 10, 20, 40, 100 and 200 ppm for 24 h. At the end of treatment cytotoxicity parameters were determined as described in the methods.

HepG2 cells were exposed to Catechin-Cu nanoparticles and Catechin at the concentration of 0, 1, 5, 10, 20, 40, 100 and 200 ppm for 24 h and cytotoxicity were determined by MTT assay. The cytotoxicity assays have shown that Catechin-Cu nanoparticles up to the concentration of 40 ppm did not produce significant cytotoxicity to cells (P > 0.05 for each), a dose that was found to be lethal for the bacteria tested in this study (Table 1). As the concentration of nanoparticles increased to 100 and 200 ppm, in MTT assay, cell viability dropped drastically to 62.1 and 59.2% respectively (Fig. 7). Furthermore, the cell viability kept about 70% in the presence of 100 ppm Catechin, a dose that the MBC of Catechin to *S. aureus*. Catechin-Cu nanoparticles show the higher cytotoxicity to HepG2 cells than that of Catechin at the concentrations range of 1–200 ppm, which can better inhibits the proliferation of HepG2 cells. Such cytotoxicity induced by Catechin-Cu nanopaticles should be attributed to the direct interaction of nanoparticle with the HepG2 cells, the result suggested that small size is a major advantage of Catechin-Cu nanopaticles.

## Discussion

There is no doubt that NPs, with its excellent adsorptivity, large specific surface area, high chemical stability and so forth, is one of the most famous stars to be used as an antimicrobial agent at present. Herein, we used a simple and low-energy-consuming approach to fabricate stable and well-dispersed Catechin-Cu nanoparticles which can not only overcome the easy-oxidation limitation of Catechin but also target the pathogenic bacterium efficiently. In our experiments, we found that the pH and temperature of reaction influence the structure of the NPs. The NPs at the concentration of 10 mg L^−1^ (10 ppm) and 20 mg L^−1^ (20 ppm) provided rapid and effective killing of up to 90% and 85% of *S. aureus* and *E. coli* within 3 h, respectively, NPs demonstrated better antibacterial efficiency than Catechin. *E. coli* presented a great resistant to the antibacterial effect of the Catechin-Cu nanoparticle in comparison to the *S. aureus* strain. This can be explained by three reasons^26^. Firstly, the absence of an outer membrane and the presence of negatively charged teichoic acid molecules within a thick peptidoglycan layer (20–80 nm) on the surface of *S. aureus* should make them more attractive to the positively charged, and more specific to be damaged by positively charged molecules than *E. coli*. Secondly, the presence of a number of small channels of porins within the outer membrane of *E. coli* may help block the entrance of the particles into the bacterial cell, making them more difficult to inhibit than *S. aureus*. Finally, the smaller dimension of *S. aureus* (sphere, i.d. ∼0.5–1 μm) may partly account for the more intimate contact with the Catechin-Cu nanoparticles, making the antibacterial activity more effective than the *E. coli* (rod, 0.3–1.0 × 1.0–6.0 μm).

**Figure 8:**
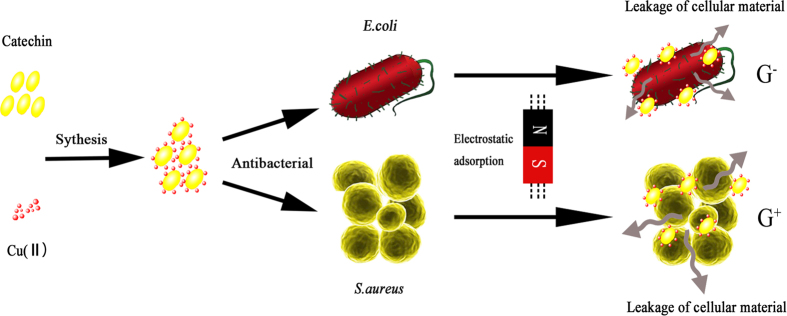
Schematic representation of antibacterial mechanism of Catechin-Cu nanoparticles. Firstly, the positive charged Catechin-Cu nanoparticles tend to be adsorbed and accumulated on the surface of negative charged cells by electrostatic attraction, which can change the permeability and enhance the antimicrobial activity. Secondly, the positive charged NPs mainly adsorb the negatively charged teichoic acid molecules in *S. aureus*, which made the NP can act on the cells effectively and fully, resulting in cell death.

Although the antibacterial effects of Catechin-Cu nanoparticles against various bacterial systems are well established, however very little is known about their bactericidal mechanism and thus mode of action. Here is an attempt to explore the antibacterial mechanism of Catechin-Cu nanoparticles on the basis of our investigation and by taking into consideration of the previous proposed findings (Fig. 8).

1. The positively charged Catechin-Cu nanoparticles should interact favorably with the negatively charged bacterial cell membrane by electrostatic attraction, causing an increase in membrane permeability and eventually rupture and leakage of intracellular components^27^,^28^ and the positive charged NPs mainly adsorb the negatively charged teichoic acid molecules in *S. aureus* (as supported by our TEM and the reason why the *S. aureus* presented a lower resistant to the antibacterial effect of the Catechin-Cu nanoparticle in comparison to the *E. coli*).
2. Disruption of the bacterial cell membrane (as supported by our SEM, TEM and AFM analysis) would be the other probable mode of nanoparticle action, as it paves the way into the bacterial cells by leading to membrane protein and lipid bilayer damage as reported in previous studies^29^,^30^. The damage could be the synergistic antibacterial effect of Catechin and copper (II) (as supported by the bacterial efficacy comparison of Catechin and Catechin-Cu nanoparticles).
3. Leakage of intracellular material due to membrane disruption may cause shrinkage of the cell membrane, ultimately leading to cellular lysis as justified by our TEM and AFM micrographs.

In summary, we have demonstrated a simple and low-energy-consuming approach to fabricate highly stable and dispersive Catechin-Cu nanoparticles by an ultrasonic cell crushing method. The results demonstrated that as-synthesized NPs displayed excellent antimicrobial activity against pathogenic bacterium *E. coli* and *S. aureus*; *E. coli* presented a great resistant to the antibacterial effect of the Catechin- Cu nanoparticle in comparison to the *S. aureus* strain because of its unique structure. However, future studies on the biocidal influence of this nanomaterial on other Gram-positive and Gram-negative bacteria are necessary to fully evaluate its possible use as a new bactericidal material. What is more, this highly dispersive Catechin-Cu nanoparticle can be easily fabricated into film and fiber, which would open an avenue for a wide range of applications of antimicrobial materials.

## Methods

### Synthesis of Catechin-Cu nanoparticles

**Figure 9:**
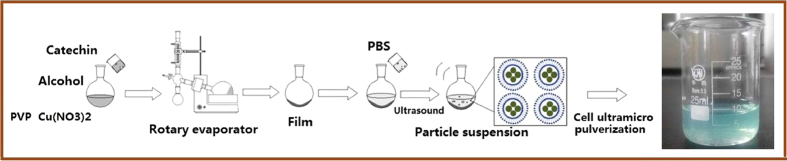
The synthetic route of Catechin-Cu nanoparticles.

Catechin-Cu nanoparticles were synthesized through an ultrasonic crushing method, as schematically depicted in Fig. 9, the major steps involved in producing Catechin-Cu nanoparticles. In a typical procedure, a solution containing 9 mL alcohol was prepared, in which a desired amount of Poly Vinyl Pyrrolidone (PVP) (Mw = 1,300,000) was dissolved with magnetic stirring, at room temperature, for 2 h. The concentration of PVP was 9% by weight, followed by addition of 1 ml of mixed solution containing Catechin and cupric nitrate with molar ratios 1:2. In this method, both the cathchin and cupric nitrate were mixed in a mutually dissolving solvent, which was removed by evaporation to produce a film, and then reconstituted in a buffer solution medium to get the particle suspension. At last, ultrasonic cell pulverization was carried out with the probe of the ultrasonic horn immersed directly into the mixture solution at 600 w for 40 min to obtain the light blue colored solution as the final nanoparticle product. There is no residual Cu^2+^ in the Catechin-Cu NPs product, which was detected by metathesis reactions of Cu^2+^ with sodium hydroxide in this work. The distinguished characteristic of PVP is its ability to be a surfactant. The products were centrifuged 10 min by using 10000 r/min centrifugal speeds and washed with deionized water and ethanol repeatedly to remove any impurities, and then the suspension was dried overnight in a vacuum oven at 60 °C to obtain Catechin-Cu NPs.

### Characterization of nanoparticles

We visualized the shape and size of the Catechin-Cu nanoparticles using a high-resolution transmission electron microscope (HR-TEM, JEOL JEM-3010). The crystal structure of the nanocomposites was characterized by a Philips 1730 powder X-ray diffractometer with CuKα radiation (λ = 1.5406 Å). The diffraction data were recorded at 6° min^−1^ between 5 and 80°. The zeta potential of Catechin-Cu nanoparticles was measured by DLS with Zerasizer Nano ZS (Malvern Instrument Ltd. Worcestershire, UK). For structure properties of NPs, the Fourier transform infrared spectroscopy (FTIR) spectra were measured using a Perkin-Elmer Spectrum GX1. The optical property of the nanoparticles was tested on a Shimadzu UV-2550 spectrophotometer at room temperature.

### Stability of nanoparticles

**Figure 10:**
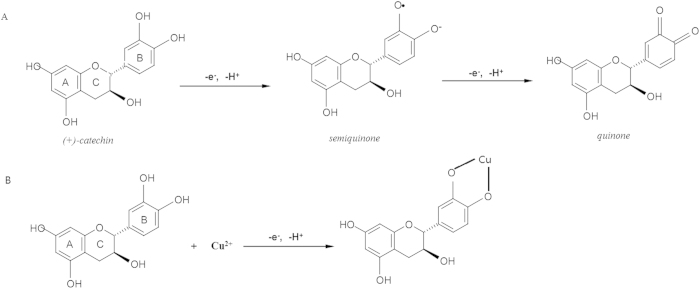
Oxidation reaction of Catechin(A), and complexation reaction between Catechin and Cu(II).

Catechin is unstably prone to the oxidation (Fig. 10A). Herein, a novel Catechin-Cu nanoparticles based on the chelating of Cu(II) to Catechin in order to overcome the demerit of Catechin (Fig. 10B). In this paper, we evaluated the stability of Catechin-Cu nanoparticles by observing dispersion of it in 10^th^, 20^th^, and 30^th^ day, the particle size of NPs and the average diameter used as the assessment indicators^31^.

### Analysis of the antibacterial activity of Catechin-Cu nanoparticles

For antibacterial tests, all plates and materials were sterilized in an autoclave before experiments. Two strains were used: *Escherichia Coli* (ATCC 8739) and *Staphylococcus aureus* CIP 65.8 T. The selected strains are typical representatives of two large bacterial taxonomic lineages and were obtained from American Type Culture Collection (ATCC, Manassas, VA, USA), Culture Collection of the Institute Pasteur (CIP, Paris, France) and the National Collection of Industrial, Food and Marine Bacteria (NCIMB, Aberdeen, UK). Prior to each bacterial attachment experiment, bacterial cultures were refreshed on nutrient agar from stocks (Oxoid, Basingstoke, Hampshire,UK). Fresh bacterial suspensions were grown overnight at 37 °C in 5 ml of nutrient broth (Oxoid, Basingstoke, Hampshire, UK). Bacterial cells were collected at the logarithmic stage of growth and the suspensions were adjusted to OD_600_ = 0.3.

### MICs and MBCs estimation

All strains were cultured routinely, harvested by centrifugation (8000 g at 4 °C for 10 min), washed twice in phosphate-buffered saline (PBS, 0.2 mol L^−1^ at pH 7.4), and resuspended at a final concentration of approximately 10^7^ cells/mL of PBS. To determine the MIC, MBC, the resuspended cells were inoculated into fresh medium supplemented with various concentrations of the NPs and grown overnight at 37 °C (or 30 °C) and then the minimum concentration of the NPs giving cultures that did not become turbid was taken to be the MIC and minimum bactericidal concentrations without any bacterial growth represented MBC. The determination of the MIC and MBC for each isolate was carried out in triplicate, and the results were taken when there was agreement in at least two of the three MIC and MBC results. Control experiments were performed with bacteria in the medium only.

### Cell viability analysis

Confocal laser scanning microscopy (CLSM) was used to visualize the proportions of live cells and dead cells using LIVE/DEAD BacLight Bacterial Viability Kit, L7012. A mixture of SYTO 9 and propidium iodide fluorescent dyes (Molecular Probes, Invitrogen, Grand Island, NY, USA)^32^. The green fluorescent dye SYTO 9 permeated both intact and damaged membranes of the cells, binding to nucleic acids and fluorescing green when excited by a 485 nm wavelength laser. On the other hand, the red fluorescent dye propidium iodide(PI) alone entered only cells with significant membrane damage, which are considered to be non-viable, and binds with higher affinity to nucleic acids than SYTO 9. Bacterial suspensions were stained according to the manufacturer’s protocol, and imaged using a Fluoview FV10i inverted microscope (Olympus, Tokyo, Japan). For spore staining, a minor modification was made to this protocol. Spores were stained with a mixture of 0.9 mmol l1^−1^ of propidium iodide and 5 mmol l1^−1^ of SYTO 9 for 15–20 min. The samples were then imaged under the confocal microscope.

### Microscopy investigation of bacteria

Bacteria were treated with the Catechin-Cu nanoparticles, then collected by centrifugation, washed twice, and resuspended in PBS. The cells were fixed for 2 h in 2.5% glutaraldehyde at room temperature (~25 °C), dehydrated, and then coated with gold. The interactions of the NPs with bacterial cells were examined using SEM. The sample preparation of transmission electron microscopy (TEM) was the same as SEM from the steps of harvest to centrifugation, and then a drop of the suspension was placed on a cooper grid and dried. Grids were conducted on a JEM-3010 TEM [JEOL Ltd., Japan] at 200 kV. Surface topography of photodynamically treated and untreated (control) bacteria were imaged using a multimode scanning probe microscope (NT-MDT, SOLVER-PRO, Russia). AFM experiments were performed by using a Veeco Multimode NS3A-02NanoscopeIII atomic force microscope. Scan size was set to obtain 2.0 μm × 2.0 μm images. Two to three areas of each bacterium were scanned using the tapping mode under nitrogen. The cantilever of the tip is a standard 115–135 μm long microlever with a force constant of 20–80 N/m and has a typical resonant frequency between 200 and 400 kHz. (SOLVER-PRO, Russia). The 3D images were obtained with the software WSxM 4.0 Develop 11.3-Package (2007, Nanotec Electronica S.L., Madrid, Spain), which was also used for image analysis. A 200 μL portion of the cell suspension was withdrawn from the above culture. The suspension was centrifuged at 4000 *g* in an Eppendorf centrifuge for 2 min. The supernatant was removed, and the cell pellet was resuspended in ice cold Tris-HCl (pH 8.0, 2 mmol/L). This procedure was repeated twice. Finally, the pellet was resuspended in Tris-HCl. A 5 μL portion of the bacteria suspension was placed on a freshly cleaved mica substrate and dried under N_2_ gas for 3 min immediately. For the topographic analysis of the bacteria with and without NPs, the parameter of surface roughness was used: the surface roughness includes mean roughness (Ra) and root-mean-square roughness (Rq). The Ra and Rq were calculated with the data of the topographical micrographs by means of the previously mentioned software and using the following expression [1], [2] and [3]^33^,^34^:


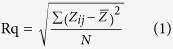



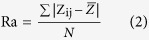



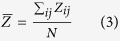


Where Z_ij_ is the height value of each single point (nm), 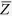 is the average height (nm) and N is the number of experimental points.

### MTT assay

Human liver HepG2 cell line was cultured in Dulbecco’s modified Eagle’s medium (DMEM) supplemented with 10% fetal bovine serum (FBS), 1% penicillin–streptomycin, which were bought from Gibco, USA. To determine the cytotoxic activity of the Catechin-Cu NPs and Catechin, human liver HepG2 cell line were seeded onto 96-well culture plates (2 × 10^4^ cells/ml), and treated with different concentrations of Catechin-Cu NPs or Catechin (0, 1, 5, 10, 20, 40, 100, 200 ppm) at 37 °C in an atmosphere of 5% CO_2_ for 24 h. After removed the cell culture medium, cells were washed with PBS twice. 3-(4,5-dimethylthiazolyl-2)-2,5- diphenyltetrazolium bromide (MTT) at a concentration of 0.5 mg/ml was added to the wells and incubated for 4 h at 37 °C in an atmosphere of 5% CO_2_ in dark. In metabolically active cells, MTT was reduced to an insoluble dark purple formazan. The formazan crystals were dissolved with DMSO. The absorbance was read at 570 nm using a plate reader and the percent of cell viability was calculated from the percent ratio of the absorbance obtained from each treatment and that of the control.

## Additional Information

**How to cite this article**: Li, H. *et al.* Enhancing the antimicrobial activity of natural extraction using the synthetic ultrasmall metal nanoparticles. *Sci. Rep.* **5**, 11033; doi: 10.1038/srep11033 (2015).

## Figures and Tables

**Table 1:** **Minimum inhibitory concentration (MIC) and minimum bactericidal concentration (MBC) of Catechin solution, Catechin-Cu nanoparticles against various microorganisms.**

**Bacteria**	**Catechin**		**Catechin-Cu nanoparticles**	
	MIC (ppm)*	MBC (ppm)**	MIC (ppm)*	MBC (ppm)**
*E. coli*	100	125	20	40
*S. aureus*	90	100	10	20

^*^Concentration (3 replications) where no turbidity was observed in culture.

^**^Concentration (3 replications) where no growth was observed on agar plate.

**Table 2:** **Effects of nanoparticles on the surface roughness of different bacteria.**

**Sample**	**Bacteria**	**Surface Roughness (nm)**	
		***Rq***	***Ra***
Untreated	*E. coli*	6.32	4.88
treated		71.6	57.3
Untreated	*S. aureus*	4.51	3.13
treated		76.1	59.9
